# Acceptance of different design exergames in elders

**DOI:** 10.1371/journal.pone.0200185

**Published:** 2018-07-05

**Authors:** Chih-Kuang Chen, Tsai-Hsuan Tsai, Yin-Chou Lin, Chung-Chih Lin, Su-Chu Hsu, Chia-Ying Chung, Yu-Cheng Pei, Alice M. K. Wong

**Affiliations:** 1 Department of Physical Medicine and Rehabilitation, Chang Gung Memorial Hospital, Taoyuan, Taiwan; 2 Healthy Aging Research Center, Chang Gung University, Taoyuan, Taiwan; 3 Department of Industrial Design, Chang Gung University, Taoyuan, Taiwan; 4 Department of Computer Science and Information Engineering, Chang Gung University, Taoyuan, Taiwan; 5 Department of New Media Art, Taipei National University of the Arts, Taipei, Taiwan; University of Illinois at Urbana-Champaign, UNITED STATES

## Abstract

For promoting the successful aging of elderly residents of Chang Gung Silver Village in Taiwan, five interactive exergames were developed to promote the well-being of the elderly. The exergames included both physical games and cognitive games, and were implemented using various computer-based technologies in the Chang Gung Silver Village. The exergames were trialed by 39 elderly residents (15 male, 24 female; mean age 79.5 ± 17.5 years) of Chang Gung Silver Village. Following the trials, the participants were requested to complete a Technology Acceptance Model 2 (TAM2) questionnaire. The results showed that the perceived playfulness and perceived usefulness of the exergames were significantly related to the users’ usage behavior and intention to use for both the physical games and the cognitive games. However, a relationship between the output quality of the game and the usage behavior was apparent only in the case of the cognitive exergames. Finally, the impact of social influence on the intention to use and the usage behavior was more pronounced for the physical exergames. Overall, the results revealed that the acceptance of exergames by the elderly depends not so much on the awareness of fun in using the game, but the perceived usefulness of the related physical and cognitive abilities.

## Introduction

According to the WHO’s “biopsychosocial model of health”, human health comprises the complete state of physical, mental and social well-being, not only the absence of disease and infirmity. Regular exercise has long been proposed as a means of enhancing the physical, mental and social functions of the elderly, thereby reducing the morbidity and mortality inherent in a sedentary lifestyle [1]. The physical benefits of exercise include reducing the risks of obesity, high blood pressure, diabetes, and even cancer [2]. Furthermore, exercise can relieve the symptoms of depression and anxiety, and improve memory and processing speed [3]. Studies have shown that aerobic exercise can also improve decision-making skills [4]. Nevertheless, older adults are sometimes reluctant to exercise due to a lack of motivation, social support and company, or a safe and practical environment in which to do so [5].

Exergames, a combination of exercise and computer game, include such commercial products as Nintendo Wii^TM^ and the XBOX Kinect^TM^. Exergames have been developing since the late 1980s. They became more commercially known thanks to DDR^TM^ (Dance Dance Revolution), a 1998 interactive dance mat on which players must move in the direction of visual cues and in time to background music. Exergames became even more popular with the success of Sony Eye Toy (2004), Nintendo Wii^®^ (2006), Wii Fit (2007), PlayStation Move^®^ (2009), and Microsoft XBOX Kinect^®^ (2010). Kinect^TM^ is the first exergame system where a camera captures body movements in real time, without the need for worn or handheld devices. Exergame has shown its potential to be a motivating and entertaining factor in assisting in rehabilitation (Rehabilitation Exercise System, IREX^TM^). It’s designed to be used by people with motor and cognitive impairments, such as patients who have suffered a stroke or traumatic brain injury, or children with cerebral palsy and learning disability.

In recent years, with advances in technology, exergames have emerged as a brand new interaction system for users, allowing them to exercise within their own homes, or in community settings. Given the specific characteristics of the elderly, many researchers have begun to investigate the potential of using exergames to improve older adults’ willingness and attitudes toward exercise. Theng and colleagues [6] assessed the efficacy of the Nintendo Wii in promoting exercise among seniors in Singapore. Bieryla and Dold [7] reported that Wii Fit training older adults could significantly increase their Berg Balance Scale (BBS) than control group. Fu and colleagues [8] developed an exergame based on Nintendo’s Wii Fit balance board to reduce the risk of falls among seniors with a history of such events. van Diest and colleagues [9] reviewed thirteen studies found that most of them reported positive results in balance ability after an exergame training period. Whitlock and colleagues [10] investigated the issue of video game usability for older adults and found that age-related changes may negatively affect their capacity to benefit from video games. Gao and Mandryk [11] found that causal exergames can yield acute cognitive benefits, as demonstrated by improved cognitive performance on tests requiring focus and attention. Harris and colleagues [12] suggested that exergames may be an appropriate therapeutic tool for improving balance and postural control in older adults. Venheijden Klompstra and colleagues [13] showed that patients with heart failure exhibited improved balance and cognitive functions after playing exergames. Overall, exergaming appears to have significant potential as an exercise strategy for older adults. Chang Gung Silver Village is a retirement community for people over 60 years of age. It services those residents who are walking and living independently without physical or cognitive impairment. We also provide them with a comprehensive physical examination including fitness, twice a year in our Chang Gung Memorial Hospital. All their physical conditions and chronic illness are supervised. The total number of dwellings in Silver Village is approximately 750, with an age range from 60 to 100 and an average age of approximately 80 years old.

The technology acceptance model (TAM) was first proposed by Davis and colleagues [14] based on the theory of reasoned action (TRA) and the theory of planned behavior (TPB) to assess a user’s behavioral intentions toward using new technologies. The TAM theorizes that an individual’s behavioral intention to use a system is determined by two beliefs, namely, perceived usefulness (PU) and perceived ease of use (PEOU). However, the original TAM was criticized for ignoring social influence and individual characteristics. Hence, an extension of the TAM, referred to as TAM2, was proposed by Venkatesh and Davis [15] that outlined perceived usefulness and usage intentions as it related to the social influence processes (subjective norms, voluntariness, and image) and cognitive instrumental processes (job relevance, output quality, result demonstrability, and perceived ease of use). In an attempt to integrate the main competing user acceptance models, Venkatesh et al. [16] further formulated Unified Theory of Acceptance and Use of Technology (UTAUT). Additionally, a TAM3 recommended by Venkatesh and Bala [17] that combined TAM2 with the model of the determinants of perceived ease of use, that presented a comprehensive nomological network of the determinants of individuals’ adoption and use of new technologies. All the aforementioned theories are based on users’ perspectives to investigate their acceptance of technological systems and effectively predict and explain their motivations and behaviors.

In recent years, the technology acceptance model (TAM, TAM2) is increasingly being applied in exploring factors that influence the willingness of older adults to use a technology, as well as their perceptions and expectations of the type of technology. For examples, the TAM2 was adopted by Tsai et al. [18] to measure user acceptance and usage of a social platform and received positive feedback from those older residents living in a senior community. With the TAM as a theoretical basis, Xue et al. [19] examined the perceived attitudes and readiness of women aged 50 years or older on adopting a mobile phone-based intervention in Singapore. Wong et al. [20] evaluated the older adults’ intention of using an Intelligent Comprehensive Interactive Care (ICIC) System using TAM2. Morevoer, Ramón-Jerónimo [21] explained the Internet use in the older segment, capturing the heterogeneity across gender in the TAM (and TAM2). Furthermore, TAM has also expanded to explore behavior patterns and intentions of older adults from the viewpoint of exergame use. Wüest et al. [22] assessed the usability and effects of an exergame-based balance and gait training program in terms of acceptance, adherence, and attrition among elderly participants by the TAM survey. Cook and Winkler [23] used TAM to explore the usability, ease of use, and enjoyment of the application of virtual worlds by older adults. Also, Ben-Sadoun et al. [24] presented the evaluation of usability and short-term training effects of X-Torp of an action Serious exerGames designed for elderly subjects with normal aging, mild and moderate cognitive impairment with regard to technology acceptance (based on the TAM). As a result, in this study, we invited the elderly residents of Chang Gung Silver Village to try the developed exergames in order to increase their levels of physical activity and improve their physiological conditions. They were also requested to answer the TAM questionnaires which were attached as Supporting Information from S1–S10 Files for these exergames.

### Hypothesis development

According to TAM2, output quality was defined as the tasks that the system was capable of performing and the degree to which those tasks could achieve their goals. This represents how well suited the system is for users in terms of those tasks [15]. The effectiveness of exergame tasks depends on providing a clear, instinctive, and usable interface for users to operate easily from the viewpoint of exercising. Therefore, we defined output quality based on user satisfaction with the interface and users’ perceptions of the product’s appearance. According to a report by Koufaris’ report [25] on the relationship between web connection quality and perceived playfulness, the higher the system quality and information quality, the more likely a user will have more fulfilling and enjoyable shopping experiences. Ahn and colleagues [26] found a positive relationship among system quality, information quality, service quality, and playfulness in online retailing. Therefore, we adopted the quality of user interaction with the system interface as the output quality of the exergame.

A relationship between quality and intention to use has been found in other studies. In the context of banking, O’Cass and Grace [27] found that if a service was evaluated as being of a higher quality, consumers would show more favorable attitudes toward that service brand. Furthermore, in the context of Internet shopping, e-service quality factors positively influence consumer attitudes toward a website. A similar result was reported by Carlson and O’Cass [28]. They concluded that with better e-service quality, users would have more positive attitudes towards a website. Accordingly, we put forth hypotheses 1 and 2.

- Hypothesis 1. There is a positive relationship between “Output quality” and “Perceived playfulness.”
- Hypothesis 2. There is a positive relationship between “Output quality” and “Usage behavior.”

The addition of playfulness is based on research by Moon and Kim’s research [29], who found that playfulness is an intrinsic belief or motive that is shaped from an individual’s experience in an environment. Perceived playfulness in this study was defined in terms of three dimensions, namely, (1) perception that one’s attention is focused on the interaction, (2) curiosity during the interaction, and (3) finding the interaction intrinsically enjoyable. Triandis and colleagues [30] proposed that positive and negative emotions might impact one’s behavior. Furthermore, based on Csikszentmihalyi's flow theory [31], in which Csikszentmihalyi defined flow as “the holistic sensation that people feel when they act with total involvement,” a positive subjective experience is a critical reason for performing an activity. In Moon and Kim’s study, positive relationships were also found between perceived playfulness and attitude toward use, and playfulness and behavioral intention to use [29]. Accordingly, we put forth hypotheses 3 and 4.

- Hypothesis 3. There is a positive relationship between “Perceived playfulness” and “Intention to use.”
- Hypothesis 4. There is a positive relationship between “Perceived playfulness” and “Usage behavior”.

Numerous studies have shown the relationship among perceived usefulness and intention to use and usage behavior. According to Kim et al., when users’ perceived usefulness of an ecommerce website is higher, they are more likely to have a positive attitude toward using it and intention to reuse it. Furthermore, Ahn et al. showed that perceived usefulness is positively related to attitude and behavioral intention to use online retailing websites [26]. In TAM2, the relationship between perceived usefulness and usage behavior was not included, unlike in TAM. Thus, we investigate the relationship between perceived usefulness and usage behavior, as outlined in hypotheses 5 and 6.

- Hypothesis 5. There is a positive relationship between “Perceived usefulness” and “Usage behavior.”
- Hypothesis 6. There is a positive relationship between “Perceived usefulness” and “Intention to use.”

In many studies based on either TAM or TAM2, the relationships between perceived ease of use and perceived usefulness and intention to use were strong, given that many studies had shown significant path between those factors. According to Kim and colleagues [32], perceived ease of use is a strong predictor of customers’ perceived usefulness and attitude toward the use of eCommerce websites. According to Ahn and colleagues, perceived ease of use positively impacted users’ perceived usefulness and attitude toward online retailing websites [26]. Thus, we investigated whether perceived ease of use affects perceived usefulness and intention using hypotheses 7 and 8.

- Hypothesis 7. There is a positive relationship between “Perceived ease of use” and “Perceived usefulness.”
- Hypothesis 8. There is a positive relationship between “Perceived ease of use” and “Usage behavior.”

In TAM2, subjective norm and image are both categorized as social influence processes [15]. Subjective norm is defined as “perceived pressures on a person to perform a given behavior and the person’s motivation to comply with those pressures” by Fishbein and Ajzen [33]. In the original TRA model, both subjective norms and attitude are related to one’s behavioral intention [34]. This was also supported by reports in Psychology Research that subjective norms are important variables that affect behavioral intention [35, 36]. For the relationship between subjective norms and the intention to use, Kim et al. [32] showed that subjective norms positively influence the attitude toward use in the context of ecommerce websites. For image, Moore and Benbasat [37] refer to it as “the degree to which one’s use of an innovation is perceived to enhance one’s status in one’s social system.” Although in TAM2, image was hypothesized to influence perceived usefulness, we investigated its relationship with intention to use and usage behavior because both image and subjective norms were seen as social influence factors. Thus, we proposed hypotheses 9, 10, 11, and 12 for social influence (image and subjective norm).

- Hypothesis 9. There is a positive relationship between “Image” and “Usage behavior.”
- Hypothesis 10. There is a positive relationship between “Image” and “Intention to use.”
- Hypothesis 11. There is a positive relationship between “Subjective norm” and “Usage behavior.”
- Hypothesis 12. There is a positive relationship between “Subjective norm” and “Intention to use.”

## Materials and methods

Before the study began, we applied to the Institutional Review Board (IRB) of the Chang Gung Medical Foundation and received the ethics committee’s approval of the study date range, from August 1, 2011 to June 30, 2015; the IRB identifier number is 100-1075B as Supporting Information S11 File. All of the participants were recruited and completed the trials during this time period, and the authors confirm that all related trials for this intervention (exergames) are registered. The ClinicalTrials.gov identifier number is NCT03084107 as Supporting Information S12 File. Before the exergames began, the consent was informed and written by the participants to know that they could discontinue the tests if discomfort developed including dizziness. We provided an assistant for each of the elderly to protect them and prevent injury.

### Participants

Thirty-nine elderly residents (15 male, 24 female) from Chang Gung Silver Village (Taiwan) participated in the study. All of them are walking and living independently without physical or cognitive impairment. In order to avoid diverse subjective discomforts of test subjects from their own physical limitations, elders with neuromuscular or musculoskeletal disease (might have pain or soreness), permanent impairment of cardio-pulmonary function (might be very easy to become fatigue or shortness of breath under the stress of test), poor balance (might be more easy to fall), frailty (might be unable to complete the whole test smoothly), and poor vision (might be difficult to interact the wall game) were excluded from this study. The mean age of the participants was 79.5 ± 17.5 years. Of the 39 participants, 1 was illiterate, 4 went to elementary school, 7 were senior high school graduates, 22 had a bachelor's degree, and 5 had a graduate degree. In terms of their frequency of computer use, 16 had never used a computer, 1 used one occasionally, 1 used one once a month, 4 used one twice a month, 7 used one once a week, 1 used one twice a week, and 9 used one every day or almost every day.

### Developed physical and cognitive exergames

In Chang Gung Silver Village, we provide an indoor space for exergames where the elderly can exercise and learn. They must first be identified and recorded by a radio-frequency identification (RFID) system in every exergame system. We designed this set of exergames for the elderly according to the guidelines of previous studies, including consideration of age-related changes in decrements in posture, balance, gait, fine motor skills, visual and auditory ability, short term memory, as well as attention and vigilance, to avoid causing any injuries [38]. One of our research staffs demonstrated these exergames and signed the consent form for publication as Supporting Information S13 File; the form stated that “The individual in this manuscript has given written informed consent (as outlined in the PLOS consent form) to publish these case details”. The DOI link: http://dx.doi.org/10.17504/protocols.io.k46cyze.

#### Interactive Wall with Life Memories

**Fig 1:**
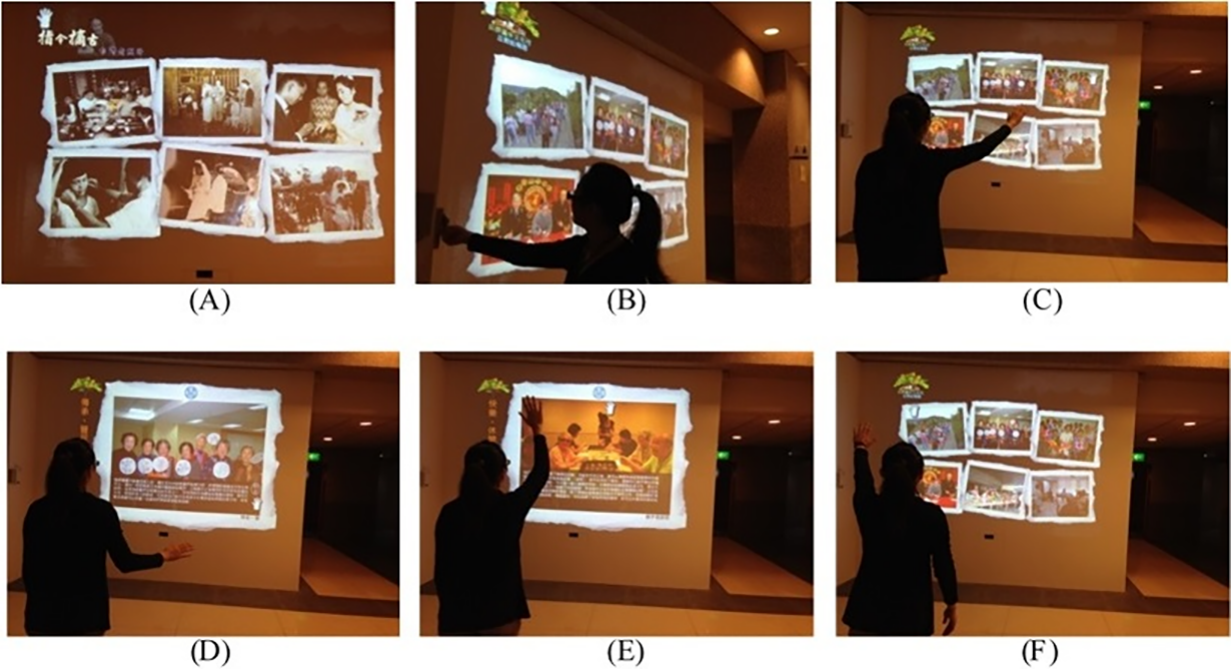
Interactive Wall with Life Memories. (A) Pictures of life memories are projected onto the wall when user stands in the interaction zone and waves their arms to initiate interaction. (B) Users employ personal RFID cards to identify themselves, and are then presented with a series of self-related photographs in the Silver Village, together with collections of pictures from former times and other educational materials. (C–F) Users employ arm gestures to navigate through various categories of photographs and, for each one, are provided with an audio commentary to facilitate the processes of reminiscence and learning.

The “Interactive Wall with Life Memories” exergame was designed to promote physical activity of the arms and upper body of the elderly, while simultaneously encouraging them to recall and share their life memories with others through the medium of photographs (Fig 1). Using the KINECT wireless sensor, the elderly stand in front of a wall projection, waving their arms to select and watch the photos of their previous activities. It involves a space interactive learning device and community learning, that is, the elderly can participate together to recollect life memories with movements of limbs and body.

#### Interactive Floor Kick and Play

**Fig 2:**
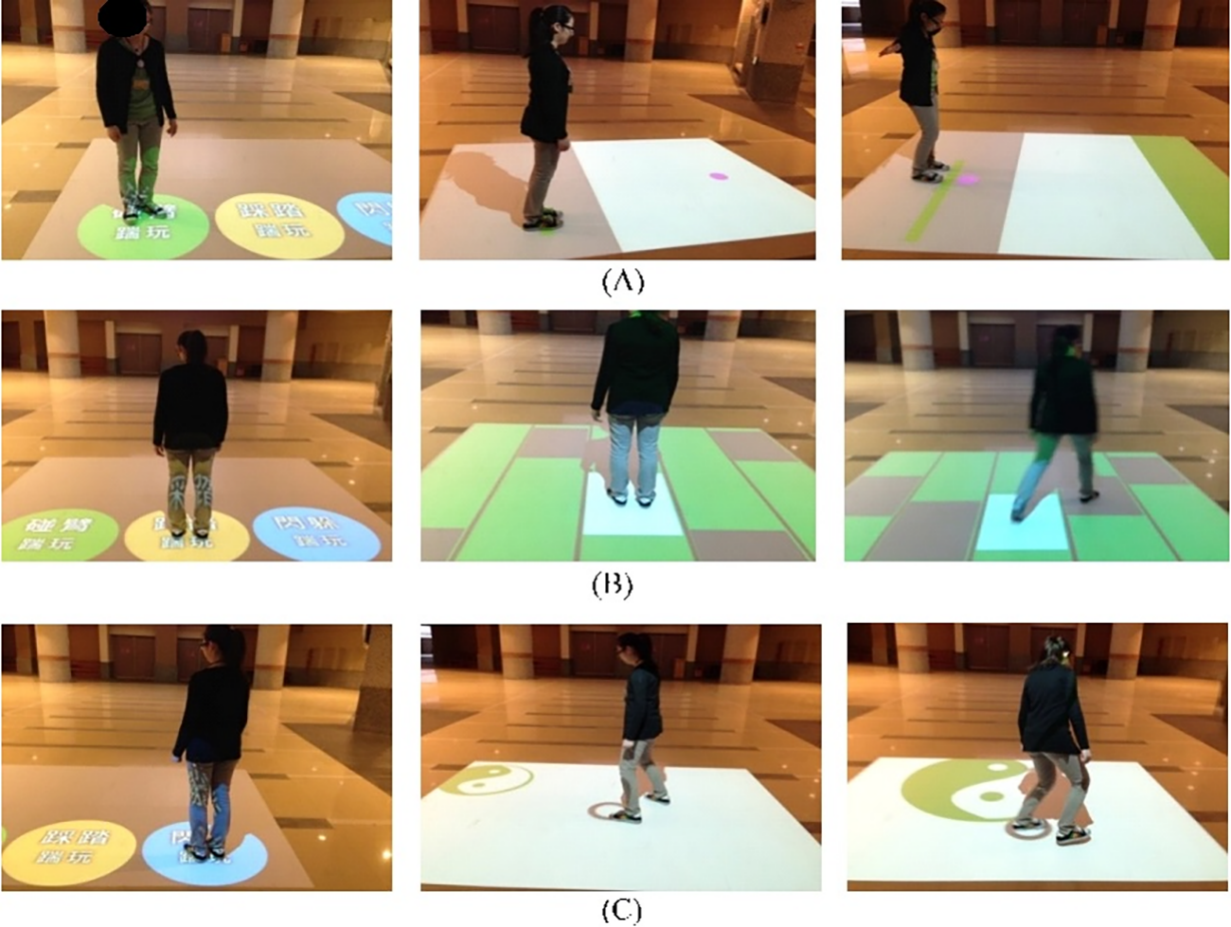
Interactive Floor Kick and Play. Three different game modes are supported: (A) Touch Arm Kick Play, (B) Stampede Kick Play, and (C) Dodging Kick Play.

The “Interactive Floor Kick and Play” game was designed to improve the reaction time and lower limb movement (with or without upper limb movement) in elderly users by responding to sudden changes in a pattern of shapes and images projected onto the floor (Fig 2). There are three games in this system at different levels of speed. The challenge in this exergame is immediate body movement in response to changes in a portion of the game area. The objective is to enhance the elderly’s physical activity and exercise response capabilities.

#### Ten Pretty Passes of the Bull

**Fig 3:**
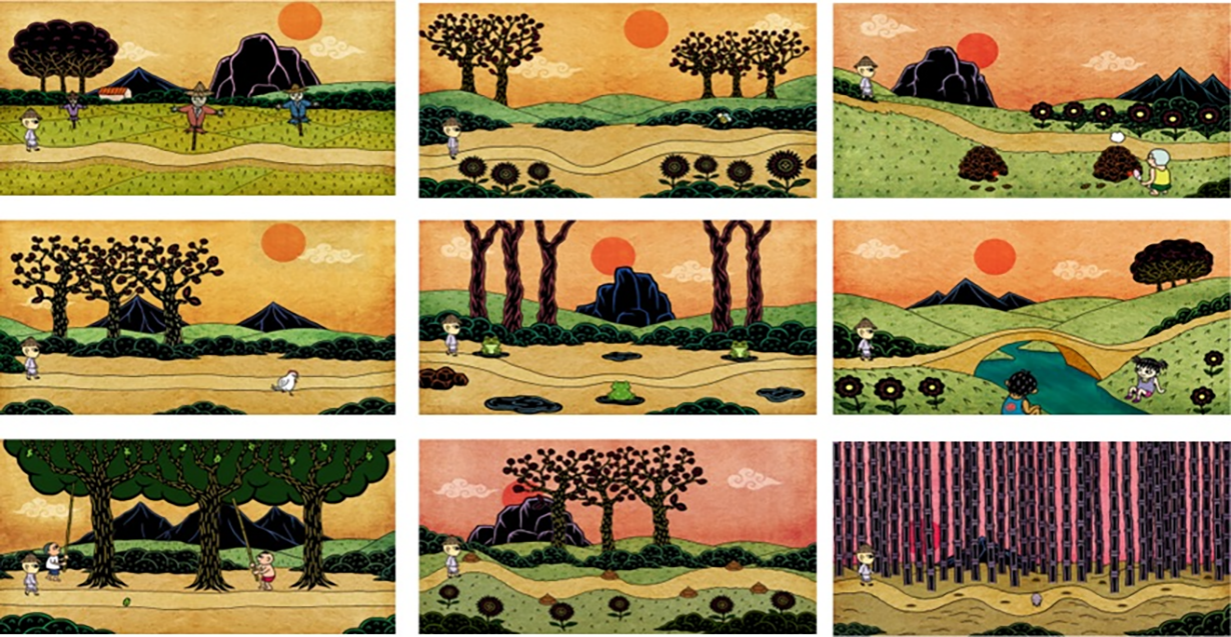
Ten Pretty Pass of the Bull.

The “Ten Pretty Passes of the Bull” game comprises a set of 10 challenges involving a cowboy and a bull presented on a touch-sensitive screen mounted on the wall (Fig 3). The aim of the game is to improve the eye-hand coordination of the users by having users react as quickly as possible to sudden events on the screen, such as a mango falling from a tree or smoke drifting from a kiln. Users are offered a choice of three difficulty levels for each station, namely, simple, ordinary and hard. If the users respond sufficiently quickly to the events they see before them, they are permitted to move onto the next challenge. At the end of the game, the participants are offered the opportunity to send a message to others via an app.

The game is set up such that users are required to take a few steps up a short uphill incline to touch the screen. Thus, in addition to improving users’ reaction time of the users, the game also increases their level of physical exertion. Moreover, the inbuilt messaging functionality reinforces social connections among the elderly in the village and their family members and friends in faraway towns.

#### Interactive Table with Musical Pots

**Fig 4:**
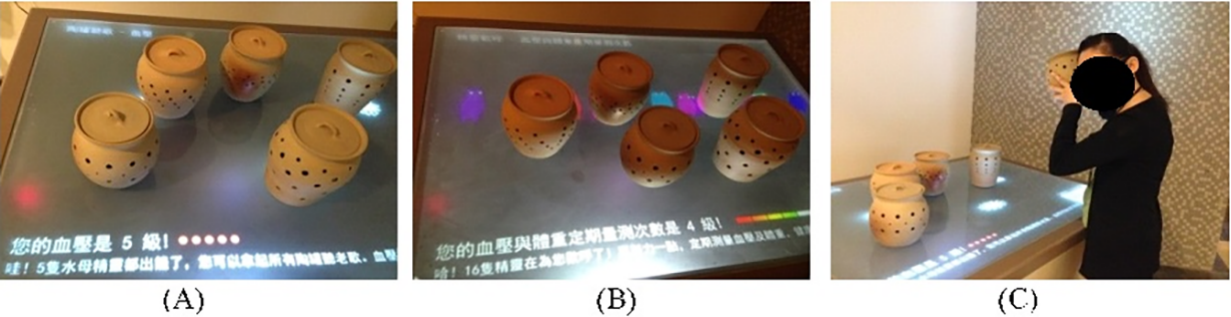
Interactive Table with Musical Pots. (A–B) After RFID identity authentication, the user is presented with a summary of their metabolic condition (C) If the medical indicator shows normal, the song within the corresponding pot plays once it is brought up to the ear.

The “Interactive Table with Musical Pots” game involves five pots placed on an interactive table and is designed to help control the metabolic status of the elderly (Fig 4). After RFID authentication, the user is presented (via the table) with a summary of their current metabolic condition in terms of five medical indicators associated with their levels of hypertension, hyperglycemia (diabetes mellitus), dyslipidemia (elevated triglyceride, high density lipoprotein (HDL)), and obesity (abdominal circumference), respectively. Each indicator is associated with a particular pot. Furthermore, each pot contains an electronic circuit and amplification system designed to play a user’s favorite song. If a medical indicator is normal, the song within the corresponding pot will play once the pot is picked up and brought to the ear. (It will then stop when the pot is returned to the table, as detected by a triaxial accelerometer.) In the event that the indicator is not normal, the song will not play when the pot is picked up. Thus, to listen to all their favorite songs, the users must control their physical condition and undergo regular physiological tests so that their indicators stored on the interactive platform can be updated. These recreational activities can also remind them to take care of their own health situation. If the pots on the table play more songs, the more favorable the health status of the elderly.

#### Fun Cube

**Fig 5:**
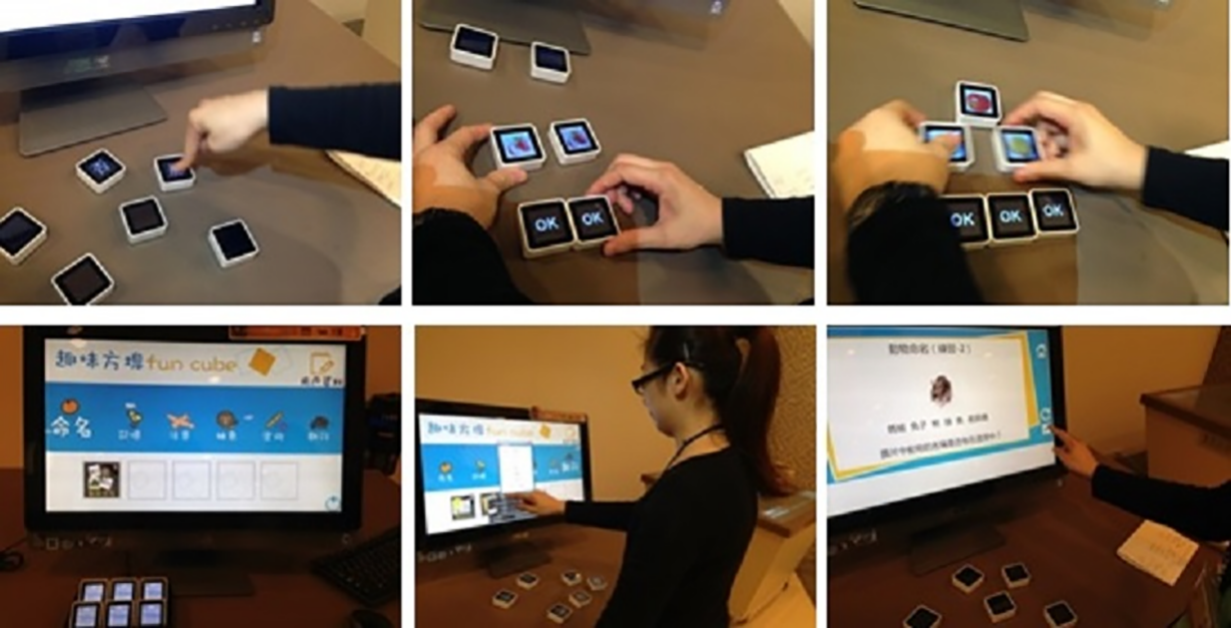
Fun Cube. The Fun cube comprises different types of games (with demonstrations) for such activities as memory training, sorting, pairing, and basic arithmetic.

The “Fun Cube” exergame involves a touch-screen computer linked to six cubes showing different pictures, figures, or words (Fig 5). The interactive screen allows the user to choose among many different games, where each game involves the use of the cubes in order to perform such tasks as memory training, sorting, pairing, and basic arithmetic. After each game is completed, the screen displays the user’s score for self-comparison purposes or for competition with others. The games are interactive, creative and challenging and are designed to fight memory loss and promote cognitive activity in a fun and user-friendly way.

The five exergames described above can be divided into two different categories, namely, ***physical*** (“Interactive Wall with Life Memories,” “Interactive Floor Kick and Play,” and “Ten Pretty Passes of the Bull”) and ***cognitive*** (“Interactive Table with Musical Pots” and “Fun Cube”).

### TAM2 questionnaire

With the TAM2 as the theoretical foundation of the research model, the experiences of the elderly in using these five exergames were self-reported using a questionnaire based on Venkatesh and Davis’s TAM2 design [15], since this model takes into account 11 variables, with four variables originating from the original TAM questionnaire (i.e. “perceived usefulness”, “perceived ease of use”, “intention to use” and “usage behavior”) and seven additional variables (“voluntariness”, “experience”, “subjective norm”, “image”, “job relevance”, “output quality” and “result demonstrability”). It it noted that, “voluntariness” and “experience” were defined as moderating variables into TAM2. “voluntariness” was defined to distinguish usage contexts into mandatory and voluntary settings. TAM2 postulated that users’ acceptance of a new system could with increasing experience. However, all of the participants were invited to participate and complete the trials in a field experiment setting at Chang Gung silver village; hence, it is not necessary to classify users’ usages into voluntary or mandatory contexts. For this reason, voluntariness was excluded from our model. Moreover, even though the elderly participants were labeled experienced or inexperienced computer users in this study, all of the respondents had no previous experiences using exergames. Thus user experience was not considered in the model because it is assumed that older adults were relatively no experienced in exergaming usage. Also, “job relevance” referred to the perception of how applicable the technology is to a user’s job. TAM2 theorized that “result demonstrability” as the perception of tangible results due to technology use. With regard to relatedness in the lives of older adults, job relevance and result demonstrability were not included in the model. That is, to achieve a better fit between the model and the present study, its semantics were modified in order to make them more consistent with the presentation of the research topics. Thus irrelevant topics in the question items were deleted, including “voluntariness”, “experience”, “job relevance”, and “result demonstrability”. Finally, an additional item of “Perceived playfulness” was added to the model. Each variable was evaluated using a seven-point Likert-type scale with anchors of 1 = strongly disagree, 2 = disagree, 3 = somewhat disagree, 4 = neither agree or disagree, 5 = somewhat agree, 6 = agree, 7 = strongly agree [13].

### Statistical analysis

To avoid older adults’ experiences in using computers affecting the results of the structural equation model, a t-test was first performed to examine whether older adults with different computer experiences would answer the questionnaire (each item and each variable as a whole) significantly differently. Participants’ responses on both physical and cognitive exergames were analyzed separately. Participants were labeled “non-experienced computer users” if they answered they never used a computer, used one occasionally, or used one once a month. Participants were labeled “experienced computer users” if they answered they used computer once every two weeks, once a week, twice a week, or at least once a day. The model used differs from the original TAM2 by adding a perceived playfulness construct as depicted in the hypothesis development section, removing voluntariness, experience, job relevance, and result demonstrability. The structural equation model was tested using Minilab and LISREL. To achieve a better fit between the model and the present study, its semantics were modified in order to make them more consistent with the presentation of the research topics. Moreover, irrelevant topics in the question items were deleted, including “voluntariness”, “experience”, “job relevance”, and “result demonstrability”. Finally, an additional item of “Perceived playfulness” was added to the model.

The reliability of the individual items with respect to the corresponding constructs was evaluated by means of the Chronbach’s Alpha coefficient. In addition, the validity of the questionnaire constructs was evaluated in terms of both the convergent validity and the discriminant validity. Convergent validity refers to the extent to which the measured variables converge with the same construct. Sufficient convergent validity requires that the average variance extracted (AVE) of the latent variables be no less than 0.5 and the latent variable composite reliability (CR) value be greater than 0.7 [13].

## Results

In the first stage of analysis, when playing physical exergames, Table 1 shows the t-test result of physical exergames between experienced and non-experienced computer users on the questionnaire items and variables. There were significant differences between the two groups of participants on perceived usefulness 1, perceived usefulness 4, perceived ease of use 1, image 1, image 2, perceived ease of use, and image; there were no significant differences on rest of the questionnaire items and variables. Unlike in the physical exergames, no significant differences were found between experienced and non-experienced computer users on all of the questionnaire items and variables of the cognitive exergams, as shown in Table 2.

**Table 1:** t-test on physical exergames between experienced and non-experienced computer users on the questionnaire items and variables.

Variable	Experience in using computer	Mean	S.D.	t-value	Significant value
Usage behavior 1	Experience	6.08	0.81	-0.29	0.77
	Non-experience	6.12	0.84		
Usage behavior 2	Experience	6.04	0.74	-0.13	0.89
	Non-experience	6.06	1.04		
Intention to use 1	Experience	5.71	1.09	-0.35	0.73
	Non-experience	5.79	1.18		
Intention to use 2	Experience	5.69	1.08	-0.69	0.50
	Non-experience	5.83	0.98		
Intention to use 3	Experience	5.00	1.40	-1.50	0.14
	Non-experience	5.40	1.22		
Perceived usefulness 1	Experience	5.08	1.41	-1.88	0.06
	Non-experience	5.58	1.25		
Perceived usefulness 2	Experience	5.48	1.44	-1.08	0.28
	Non-experience	5.77	1.24		
Perceived usefulness 3	Experience	5.38	1.29	-0.68	0.50
	Non-experience	5.56	1.35		
Perceived usefulness 4	Experience	5.40	1.38	-1.91	0.06
	Non-experience	5.88	1.08		
Perceived ease of use 1	Experience	5.81	0.89	2.58	0.01
	Non-experience	5.08	1.75		
Perceived ease of use 2	Experience	5.73	0.84	1.01	0.31
	Non-experience	5.52	1.19		
Perceived ease of use 3	Experience	5.77	0.85	1.15	0.25
	Non-experience	5.54	1.11		
Perceived playfulness 1	Experience	5.90	0.89	-0.63	0.53
	Non-experience	6.02	0.96		
Perceived playfulness 2	Experience	5.92	0.86	-0.07	0.94
	Non-experience	5.94	1.16		
Perceived playfulness 3	Experience	5.63	1.10	-0.14	0.89
	Non-experience	5.67	1.24		
Subjective norm 1	Experience	5.38	1.07	-0.63	0.53
	Non-experience	5.52	1.09		
Subjective norm 2	Experience	5.19	1.16	-1.38	0.17
	Non-experience	5.50	1.07		
Image 1	Experience	5.33	1.40	-3.07	0.00
	Non-experience	6.02	0.81		
Image 2	Experience	5.58	1.16	-1.94	0.06
	Non-experience	5.98	0.91		
Output quality 1	Experience	5.85	0.85	-0.05	0.96
	Non-experience	5.85	0.95		
Output quality 2	Experience	5.83	0.83	0.31	0.75
	Non-experience	5.77	0.95		
Usage behavior	Experience	6.06	0.75	-0.22	0.82
	Non-experience	6.09	0.86		
Intention to use	Experience	5.47	1.11	-0.96	0.34
	Non-experience	5.67	1.01		
Perceived usefulness	Experience	5.33	1.16	-1.65	0.10
	Non-experience	5.70	1.03		
Perceived ease of use	Experience	5.77	0.80	2.03	0.05
	Non-experience	5.38	1.07		
perceived playfulness	Experience	5.82	0.83	-0.30	0.77
	Non-experience	5.88	1.01		
Subjective norm	Experience	5.29	1.08	-1.06	0.29
	Non-experience	5.51	1.01		
Image	Experience	5.45	1.21	-2.72	0.01
	Non-experience	6.00	0.78		
Output quality	Experience	5.84	0.78	0.14	0.89
	Non-experience	5.81	0.91		

**Table 2:** t-test on cognitive exergames between experienced and non-experienced computer users on the questionnaire items and variables.

Variable	Experience in using computer	Mean	S.D.	t-value	Significant value
Usage behavior 1	Experience	5.26	1.50	-0.89	0.38
	Non-experience	5.53	0.97		
Usage behavior 2	Experience	5.43	1.24	-0.02	0.99
	Non-experience	5.43	1.19		
Intention to use 1	Experience	5.09	1.58	-1.04	0.30
	Non-experience	5.43	1.10		
Intention to use 2	Experience	4.63	1.72	-1.61	0.11
	Non-experience	5.27	1.44		
Intention to use 3	Experience	4.29	1.56	-0.57	0.57
	Non-experience	4.50	1.48		
Perceived usefulness 1	Experience	4.51	1.54	-0.14	0.89
	Non-experience	4.57	1.46		
Perceived usefulness 2	Experience	5.17	1.58	-0.43	0.67
	Non-experience	5.33	1.40		
Perceived usefulness 3	Experience	5.34	1.31	-1.44	0.16
	Non-experience	5.73	0.87		
Perceived usefulness 4	Experience	5.23	1.44	-0.62	0.54
	Non-experience	5.43	1.17		
Perceived ease of use 1	Experience	5.26	1.20	1.53	0.13
	Non-experience	4.70	1.66		
Perceived ease of use 2	Experience	5.40	1.09	0.38	0.71
	Non-experience	5.27	1.66		
Perceived ease of use 3	Experience	5.34	1.19	0.51	0.61
	Non-experience	5.17	1.58		
Perceived playfulness 1	Experience	5.14	1.54	-1.30	0.20
	Non-experience	5.60	1.25		
Perceived playfulness 2	Experience	5.63	1.19	-0.37	0.72
	Non-experience	5.73	1.11		
Perceived playfulness 3	Experience	5.11	1.64	-1.26	0.21
	Non-experience	5.57	1.17		
Subjective norm 1	Experience	4.89	1.43	-0.47	0.64
	Non-experience	5.03	1.07		
Subjective norm 2	Experience	4.86	1.38	-0.03	0.97
	Non-experience	4.87	0.97		
Image 1	Experience	5.34	1.31	-0.83	0.41
	Non-experience	5.60	1.16		
Image 2	Experience	5.43	1.24	-0.71	0.48
	Non-experience	5.63	1.07		
Output quality 1	Experience	5.60	1.04	0.40	0.69
	Non-experience	5.50	0.97		
Output quality 2	Experience	5.37	1.29	-0.09	0.93
	Non-experience	5.40	1.25		
Usage behavior	Experience	5.34	1.29	-0.49	0.63
	Non-experience	5.48	0.98		
Intention to use	Experience	4.67	1.50	-1.18	0.24
	Non-experience	5.07	1.20		
Perceived usefulness	Experience	5.06	1.31	-0.67	0.50
	Non-experience	5.27	1.07		
Perceived ease of use	Experience	5.33	1.08	0.93	0.36
	Non-experience	5.04	1.43		
perceived playfulness	Experience	5.30	1.28	-1.13	0.27
	Non-experience	5.63	1.12		
Subjective norm	Experience	4.87	1.38	-0.27	0.79
	Non-experience	4.95	0.94		
Image	Experience	5.39	1.25	-0.79	0.43
	Non-experience	5.62	1.08		
Output quality	Experience	5.49	1.05	0.14	0.89
	Non-experience	5.45	0.99		

### Measurement model

The measurement model was then analyzed for physical and cognitive exergames. First, the Cronbach’s Alpha coefficient, which was used to test the reliability of individual items with respect to the corresponding construct variables, as well as the validity of the questionnaire’s constructs, was obtained. For both types of exergames, Cronbach’s alpha values of all items were higher than 0.7, and the item-total correlations of each item exceeded 0.3, thus satisfying the research criteria. For physical exergames, the relevant coefficients of the individual items and the item-total correlations of each dimension were all higher than 0.6, while those of cognitive exergames were higher than 0.8, indicating that the instrument had a high degree of reliability and the measured results could be considered very stable. Factor loading was also obtained by conducting confirmatory factor analysis. According to Hair et al., for sample sizes of 100 and 70, factor loading should be at least 0.55 and 0.65, respectively, for a factor to be considered reliable [39]. The results are shown in Table 3 and Table 4. Since the sample size of the tests for the three physical exergames was 103, we used the criterion 0.55 and found that all items’ factor loading surpassed the threshold, except for PU1 in perceived usefulness, which was close to 0.55. For the tests of the two cognitive exergames, the sample size was 66; hence, we selected 0.65 as the screening criteria. According to the results, all items’ factor loading surpassed the threshold.

**Table 3:** Reliability and validity of measurement model for physical exergames.

Variable	Item	Item reliability (Cronbach’s α value)	Factor loading	Composite reliability	Average variance extracted	Item-Total Correlation
Intention to use	IU1	0.9419	0.930	0.855	0.749	0.926
	IU2	0.9424	0.790			0.937
Usage behavior	UB1	0.9397	0.910	0.902	0.755	0.918
	UB2	0.9395	0.880			0.908
	UB3	0.9403	0.820			0.911
Perceived usefulness	PU1	0.9437	0.510	0.876	0.649	0.680
	PU2	0.9399	0.850			0.879
	PU3	0.9401	0.840			0.861
	PU4	0.9381	0.950			0.923
Perceived ease of use	EOU1	0.9495	0.550	0.826	0.624	0.822
	EOU2	0.9472	0.830			0.839
	EOU3	0.9466	0.940			0.871
Perceived Playfulness	PP1	0.94	0.890	0.880	0.710	0.902
	PP2	0.9396	0.880			0.901
	PP3	0.9416	0.750			0.871
Subjective norm	SN1	0.9419	0.880	0.907	0.830	0.953
	SN2	0.9413	0.940			0.957
Image	I1	0.9423	0.800	0.857	0.751	0.942
	I2	0.9399	0.930			0.926
Output quality	OP1	0.9411	0.890	0.885	0.794	0.947
	OP2	0.9406	0.890			0.947

**Table 4:** Reliability and validity of measurement model for cognitive exergames.

Variable	Item	Item reliability (Cronbach’s α value)	Factor loading	Composite reliability	Average variance extracted	Item-Total Correlation
Intention to use	IU1	0.9517	0.800	0.827	0.706	0.925
	IU2	0.9498	0.880			0.919
Usage behavior	UB1	0.9501	0.870	0.898	0.747	0.890
	UB2	0.9499	0.910			0.942
	UB3	0.9508	0.810			0.899
Perceived usefulness	PU1	0.9511	0.720	0.913	0.778	0.847
	PU2	0.9499	0.900			0.914
	PU3	0.9509	0.800			0.854
	PU4	0.949	0.950			0.928
Perceived ease of use	EOU1	0.9579	0.690	0.886	0.726	0.852
	EOU2	0.9553	0.980			0.932
	EOU3	0.9545	0.860			0.910
Perceived Playfulness	PP1	0.9497	0.930	0.898	0.690	0.945
	PP2	0.9498	0.910			0.898
	PP3	0.9517	0.740			0.873
Subjective norm	SN1	0.952	0.920	0.924	0.859	0.967
	SN2	0.9522	0.930			0.963
Image	I1	0.9514	0.930	0.961	0.925	0.981
	I2	0.9508	0.990			0.979
Output quality	OP1	0.952	0.750	0.776	0.635	0.882
	OP2	0.9509	0.840			0.921

**Table 5:** Discriminant validity of the latent constructs for physical exergames.

	Perceived ease of use	Image	Intention to use	Output quality	Perceived playfulness	Perceived usefulness	Subjective norm	Usage behavior
Perceived ease of use	**0.846**							
Image	.195	**0.931**						
Intention to use	.225	.455	**0.931**					
Output quality	.267	.666	.577	**0.946**				
Perceived Playfulness	.245	.598	.714	.748	**0.892**			
Perceived usefulness	.171	.743	.630	.701	.777	**0.849**		
Subjective norm	.141	.661	.360	.626	.603	.693	**0.953**	
Usage behavior	.196	.678	.706	.620	.799	.820	.522	**0.919**

Note: The diagonals represent the average variance extracted (AVE), while the other matrix entries represent the shared variance (the squared correlations).

** P<0.01

* P<0.05.

**Table 6:** Discriminant validity of the latent constructs for cognitive exergames.

	Perceived ease of use	Intention to use	Image	Output quality	Perceived Playfulness	Perceived usefulness	Subjective norm	Usage behavior
Perceived ease of use	**0.893**							
Intention to use	.292	**0.921**						
Image	.224	.528	**0.979**					
Output quality	.402	.735	.657	**0.899**				
Perceived Playfulness	.307	.859	.586	.726	**0.905**			
Perceived usefulness	.282	.815	.768	.673	.806	**0.886**		
Subjective norm	.367	.382	.690	.562	.563	.545	**0.964**	
Usage behavior	.260	.770	.639	.611	.805	.863	.541	**0.911**

Note: The diagonals represent the average variance extracted (AVE), while the other matrix entries represent the shared variance (the squared correlations).

** P<0.01

* P<0.05.

Efficiency analysis was conducted using convergent validity and discriminant validity. Convergent validity refers to the extent to which measured variables converge with the same construct. Sufficient convergent validity requires that the average variance extracted (AVE) of latent variables be no less than 0.5 and the latent variable composite reliability (CR) value be greater than 0.7. Both types of exergames’ AVE values are higher than the threshold value of 0.5, and the latent variable composite reliability values are higher than 0.7, indicating that each construct in the measurement tool has good convergent validity. Furthermore, a measurement model should be capable of providing discriminant validity, where the square root of the AVE of the potential change items must be greater than the correlation coefficient of the other dimensions. The results showed that the square root of the average change in the latent variables was greater than the correlation coefficient of the other dimensions, thus confirming good discriminant validity of the constructs used in this study, as seen in Table 5 and Table 6. In summary, the measurement model tests, including convergent validity and discriminant validity, were satisfactory.

In terms of the fitness measures of the measurement model, Hair [40] indicated that seven common fitness measures were used to test the measurement model fit, including chi-square/degree of freedom (X2/df), goodness-of-fit index (GFI), adjusted goodness-of-fit index (AGFI), normalized fit index (NFI), non-normalized fit index (NNFI), comparative fit index (CFI), and root mean square error of approximation (RESEA). In terms of the fitness of the measurement model for physical exergames, with the exception of the AGFI value of 0.72, which is lower than the threshold value of 0.8, the rest of the indexes meet satisfy the threshold, as shown in Table 7. For the cognitive exergames, GFI, AGFI, and RMSEA failed to meet the threshold value, while the rest of the indexes satisfied the threshold. Based on the result, the fitness measures of the measurement model for physical exergames were better than those for cognitive exergames, as shown in Table 7. We assume this was because the sample size associated with the physical exergames was larger than that associated with the cognitive exergames, which might have affected the result of the fitness measures for the measurement models.

**Table 7:** Fit indexes for the measurement model of both types of exergames.

Measures	Recommended criteria	Suggested by authors	Physical exergames	Cognitive exergames
χ2/df	< 3.0	Bentler [41]	1.733	2.236
GFI	> 0.8	Seyal [42]	0.810	0.690
AGFI	> 0.8	Scott [43]	0.720	0.550
NFI	> 0.9	Hair Jr [40]	0.950	0.910
NNFI	> 0.9	Hair Jr [40]	0.970	0.930
CFI	> 0.9	Bagozzi [44]	0.980	0.950
RMSEA	< 0.08	Bagozzi [44]	0.075	0.120

### Structural model

**Fig 6:**
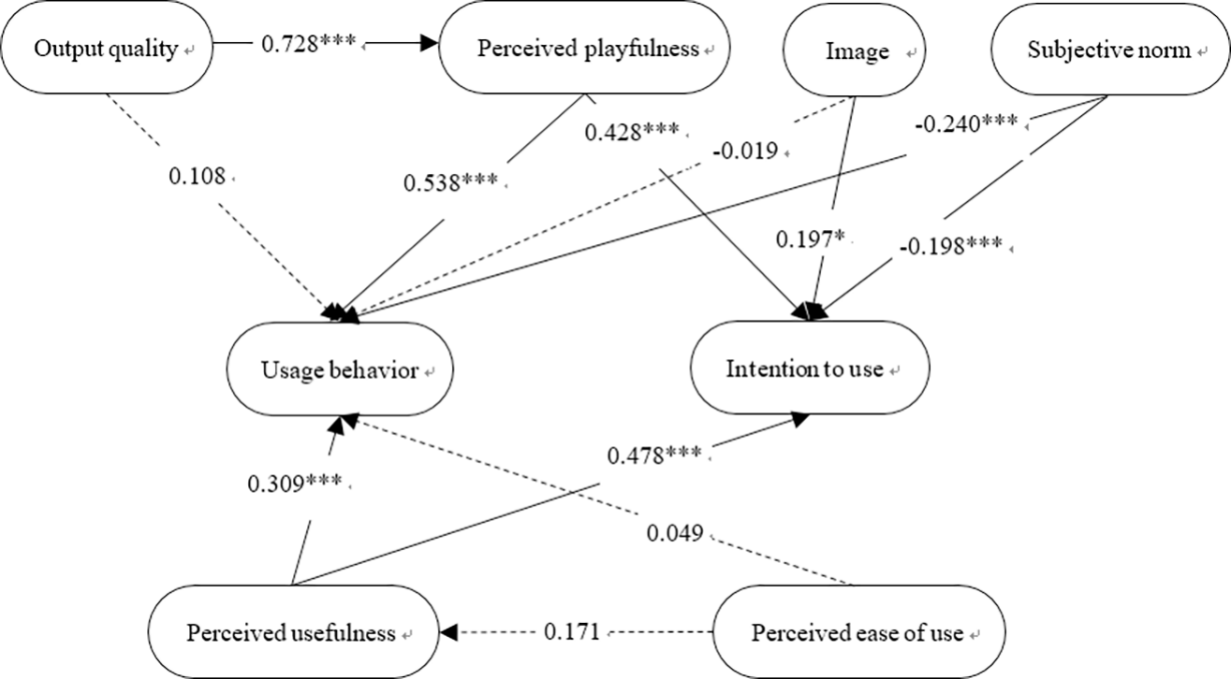
Path analysis model for research hypotheses pertaining to physical exergames.

**Fig 7:**
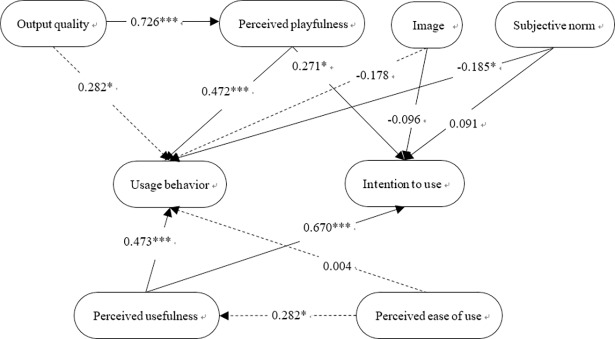
Path analysis model for research hypotheses pertaining to cognitive exergames.

The hypotheses underlying the conceptual framework of the study were tested using a structural equation model. The path coefficients and statistics of the 12 hypotheses are shown in Fig 6 and Table 8 for the physical exergames and Fig 7 and Table 9 for the cognitive exergames.

**Table 8:** TAM2 analysis of research hypotheses pertaining to physical exergames.

Extrinsic variables	→	Intrinsic variables	Regression coefficients	t-value	Significance	Path analysis
H1.Output quality	→	Perceived playfulness	0.728	9.20	***	Supported
H2.Output quality	→	Usage behavior	0.108	0.53	0.597	Not supported
H3. Perceived playfulness	→	Intention to use	0.428	6.24	***	Supported
H4. Perceived playfulness	→	Usage behavior	0.538	4.18	***	Supported
H5. Perceived usefulness	→	Usage behavior	0.309	3.52	***	Supported
H6. Perceived usefulness	→	Intention to use	0.478	4.77	***	Supported
H7. Perceived ease of use	→	Perceived usefulness	0.171	1.92	0.057	Not supported
H8. Perceived ease of use	→	Usage behavior	0.049	0.25	0.803	Not supported
H9. Image	→	Usage behavior	-0.019	-0.04	0.968	Not supported
H10. Image	→	Intention to use	0.197	2.01	*	Supported
H11. Subjective norm	→	Usage behavior	-0.240	-3.34	***	Supported
H12.Subjective norm	→	Intention to use	-0.198	-3.42	***	Supported

*** p<0.001

** p<0.01

* p<0.05

**Table 9:** TAM2 analysis of research hypotheses pertaining to cognitive exergames.

Extrinsic variables	→	Intrinsic variables	Regression coefficients	t-value	Significance	Path analysis
H1.Output quality	→	Perceived playfulness	0.726	9.76	***	Supported
H2.Output quality	→	Usage behavior	0.282	2.62	*	Supported
H3. Perceived playfulness	→	Intention to use	0.271	1.99	*	Supported
H4. Perceived playfulness	→	Usage behavior	0.472	3.95	***	Supported
H5. Perceived usefulness	→	Usage behavior	0.473	3.79	***	Supported
H6. Perceived usefulness	→	Intention to use	0.670	4.53	***	Supported
H7. Perceived ease of use	→	Perceived usefulness	0.282	2.33	*	Supported
H8. Perceived ease of use	→	Usage behavior	0.004	0.05	0.960	Not supported
H9. Image	→	Usage behavior	-0.178	-1.70	0.090	Not supported
H10. Image	→	Intention to use	-0.096	0.84	0.403	Not supported
H11. Subjective norm	→	Usage behavior	-0.185	-2.28	*	Supported
H12.Subjective norm	→	Intention to use	0.091	0.96	0.340	Not supported

*** p<0.001

** p<0.01

* p<0.05

## Discussion

According to the t-test results as shown in Table 1 and Table 2, although there were a few significant differences on different computer experiences older adults’ response to the questionnaire on physical exergames, the mean values differences were less than one (e.g. the mean value for experience and non-experience participants on perceived usefulness 1 were respectively 5.08 and 5.58). Furthermore, there were no significant differences on the questionnaire responses on cognitive exergames. Hence, we believed that computer experiences will not affect the SEM results of the study. van Diest and colleagues [9] reported the study of system review in exergames for balance training of elderly and found that exergames held interesting opportunities for improvement of balance in those elderly with higher motivation and enjoyment. Anderson-Hanley and colleagues [45] also found that an exergame of cyber cycling for the elderly achieved better cognitive function than did traditional exercise.

This set of exergames was designed to create a space with physical and cognitive exercise through art and technology in social interaction for the health promotion of the elderly. In designing the exergames, it was speculated that different types of games may result in differing degrees of acceptance and impact among the elderly. Accordingly, as described above, two specific types of games were designed, i.e., physical games (“Interactive Wall with Life Memories,” “Interactive Floor Kick and Play,” and “Ten Pretty Passes of the Bull”) and cognitive exergames (“Interactive Table with Musical Pots” and “Fun Cube”).

For both types of exergames, the path analysis results revealed the existence of a significant relationship between the output quality variable and the perceived playfulness variable. In other words, it appears that if users are satisfied with the exergame interface and its appearance, they find the exergame more interesting and enjoyable. This inference is consistent with that reported by Koufaris [25], that the user enjoyment experienced when using a particular website is directly related to the quality of the information and services which it provides. However, a relationship between the output quality variable and the usage behavior variable was found only in the case of the cognitive exergames. This suggests that cognitive exergames require the user to focus more carefully on the game interface in order to acquire the information needed to play the games and achieve task completion. In other words, interface design is more important for cognitive exergames than for physical exergames.

For both types of exergames, both the perceived playfulness and perceived usefulness variables were both related to the intention and usage behavior variables. In other words, it appears that when users both enjoys playing an exergame and recognizes its health benefits, they cultivate a more positive attitude toward the game, which is reflected in a greater intention to use the game and a more proactive usage behavior. This result is consistent with that of Moon and Kim [29]. The findings regarding the perceived usefulness variable are also consistent with those presented in [26, 32], which show that when users perceive an e-commerce website to have a higher usefulness, they are more likely to have a positive attitude toward accessing the site and to reusing it in the future.

A positive relationship was not found between the perceived ease of use variable and the usage behavior variable in either type of exergame. Furthermore, a relationship between the perceived ease of use and the perceived usefulness was found only for the cognitive exergames. This somewhat surprising result may stem from users in this study having only limited prior experience in using computer technology. For example, nearly half of the users (16 people) reported that they had never used a computer before. As a result, the exergames represent a very new experience for many of them, and it seems that this affects both the perceived usefulness of the games and the behavior of the users when actually playing the physical exergames.

For the physical exergames, the image and subjective norm variables were both found to affect the users’ intention to use. Furthermore, the subjective norm variable also affected their usage behavior. In other words, the physical exergames encourage the users to focus on their health and promote a willingness to play them. Moreover, the use of a physical exergame by peers and friends also influences their willingness to use the game and enhances their positive feelings toward it. For cognitive exergames, the subjective norm again influences the user’s usage behavior. However, no significant relationships were found between the subjective norm and the intention to use variables, or between the image variable and the usage behavior or intention to use variables.

Overall, the results of this study show that perceived playfulness is significantly related to the usage behavior and intention to use of elderly users for both physical and cognitive exergames. In addition, the output quality of the game affects the perceived playfulness and usage behavior of the elderly for both types of games. As for the social influence effect on the usage behavior and intention to use characteristics of elderly users, it appears that when the games are played in a public space and involve obvious physical movement, they attract the attention of other residents in Silver Village, and hence lead to increased social interaction under the premise of health promotion. This assertion may well explain the greater acceptance of the physical exergames compared to the cognitive exergames, which are generally more static in nature.

Around half of the users in the present study reported having no previous experience in using computer technology products. Table 1 shows the t-test result of physical exergames between experienced and non-experienced computer users on the questionnaire items and variables. There were significant differences between the two groups of participants on perceived playfulness 1, perceived playfulness 4, perceived ease of use 1, image 1, image 2, perceived ease of use, and image; there were no significant differences on rest of the questionnaire items and variables. Unlike the physical exergames, no significant differences were found between experienced and non-experienced computer users on all of the questionnaire items and variables of the cognitive exergames, as shown in Table 2. The results suggest that this affects their perceived ease of use in playing the exergames. It may also account for the absence of any significant relationship between the perceived ease of use and the usage behavior (for both types of exergames), or the perceived ease of use and the perceived usefulness (for the physical exergames). The present results have found a generally positive relationship between the social influence aspects of physical exergames and the intention to use and usage behavior of the users. However, similar relationships were not observed for the cognitive exergames. Thus, future studies should attempt to improve the design of cognitive exergames in such a way as to attract the attention of others and foster in them an interest in trying the games for themselves.

## Conclusions

The results of this study show that the perceived playfulness and perceived usefulness of physical and cognitive exergames are significantly related to the usage behavior and intention to use of elderly users. However, the output quality affects the usage behavior only in the case of cognitive exergames. In addition, social influence affects the intention to use and usage behavior of elderly users more strongly for physical exergames than for cognitive exergames. Overall, the results of this study suggest that in designing exergames for the elderly, perceived playfulness and perceived usefulness are two main factors that affect their willingness to use exergames. Designers should also bear in mind that when designing physical exergames, image and subjective norms are also two variables that could affect users’ intention to play physical exergames.

## Supporting information

S1 File TAM of Interactive Wall with Life Memories_Cht. (PDF)

S2 File TAM of Interactive Wall with Life Memories_Eng. (PDF)

S3 File TAM of Interactive Floor Kick and Play_Cht. (PDF)

S4 File TAM of Interactive Floor Kick and Play_Eng. (PDF)

S5 File TAM of Ten Pretty Passes of the Bull_Cht. (PDF)

S6 File TAM of Ten Pretty Passes of the Bull_Eng. (PDF)

S7 File TAM of Interactive Table with Musical Pots_Cht. (PDF)

S8 File TAM of Interactive Table with Musical Pots_Eng. (PDF)

S9 File TAM of Fun Cube_Cht. (PDF)

S10 File TAM of Fun Cube_Eng. (PDF)

S11 File 100-1075B IRB Eng. (PDF)

S12 File ClinicalTrial NCT03084107. (PDF)

S13 File Consent form for publication. (PDF)
