# Supplementary material for: Acceptance of different design exergames in elders
Source: PLoS One. 2018 Jul 5;13(7):e0200185. doi: 10.1371/journal.pone.0200185 (PMC6033453; doi:10.1371/journal.pone.0200185)
Supplement: S1 File — (PDF) [file pone.0200185.s001.pdf]

互動科技藝術裝置之科技接受模式問卷

敬愛的 受測者 您好：

本問卷為長庚醫院、長庚大學及台北藝術大學合作研究計畫之研究工具，問卷參考自 2000 年由 Venkatesh 和 Davis 所發展的科技接受模式 2 問卷(TAM2)。其目的是希望了解您對於 生 命 記 憶 互 動 牆 （設備）的**使用及接受情形**。

感謝您撥冗指教！

敬頌 研祺

研究者　：　桃園長庚醫院復健科敬上

聯絡電話：　(03) 328-6200 # 2381

聯絡地址：　333 桃園縣龜山鄉復興街 5 號

受測者基本資料

姓名　　：___________________________

性別　　：___________________________

年齡　　：___________________________

教育程度：___________________________

電腦使用頻率：____從不使用　____每天至少一次　____每週一次　____每兩週一次　____每月一次

聯絡方式：住處電話 _________________________　手機 _________________________

	題目內容	非常同意	同意	有點同意	沒有意見	有點不同意	不同意	非常不同意	建議(可以不填寫)
使用態度	使用『生命記憶互動牆』是一種良好的休閒活動。								
	使用『生命記憶互動牆』是一種具有正面效益的活動。								
使用意願	若將來有機會，我想我會使用『生命記憶互動牆』。								
	若我有空時，我將會經常使用『生命記憶互動牆』。								
	我會把使用『生命記憶互動牆』視為生活的一部份。								
認知有用性	『生命記憶互動牆』有助於我瞭解回想起生活的點滴。								
	『生命記憶互動牆』會促進我用腦思考。								
	『生命記憶互動牆』有助於我瞭解自己的健康狀況。								
	我有發覺，『生命記憶互動牆』對我的身心健康有幫助。								
認知易用性	將來使用『生命記憶互動牆』的時後，我不需要額外的協助。								
	學習如何使用『生命記憶互動牆』，對我來說很容易。								
	我有發覺,記住如何使用『生命記憶互動牆』是很容易的。								

	題目內容	非常同意	同意	有點同意	沒有意見	有點不同意	不同意	非常不同意	建議(可以不填寫)
認知趣味性	使用『生命記憶互動牆』後，我覺得生命記憶互動牆很有趣。								
	我實際使用『生命記憶互動牆』的過程是愉快的。								
	使用『生命記憶互動牆』會引起我的好奇心。								
主觀規範	使用過『生命記憶互動牆』之後，我認為其他住民也應該要使用『生命記憶互動牆』。								
	如果有機會使用的話，我認為我重要的親友應該也要使用『生命記憶互動牆』。								
主觀印象	據我所知，使用『生命記憶互動牆』的人，都是很重視健康的人。								
	我覺得使用『生命記憶互動牆』是個先進的健康概念。								
產出品質	整體來說，『生命記憶互動牆』的藝術造型令我滿意。								
	我對於『生命記憶互動牆』的品質感到滿意。								
效果論證	使用『生命記憶互動牆』後，我樂於告訴其他人它的好處。								
	使用『生命記憶互動牆』後，我會跟其他人交換使用心得。								

對於生命記憶互動牆，您是否還有其他意見或建議？
