# Supplementary material for: Acceptance of different design exergames in elders
Source: PLoS One. 2018 Jul 5;13(7):e0200185. doi: 10.1371/journal.pone.0200185 (PMC6033453; doi:10.1371/journal.pone.0200185)
Supplement: S2 File — (PDF) [file pone.0200185.s002.pdf]

Technology Acceptance Model Questionnaire of Interactive Wall with Life Memories.

Researcher:

Date:

Section One: Background Information:

[1] Gender ☐ Male ☐ Female

[2] Age: ______________ years old

[3] Educational Level:

☐ Elementary School ☐ Junior High ☐ Senior High ☐ Bachelor's Degree

☐ Graduate Degree

[4] Have you ever used a computer?

☐ Yes (If you answered Yes, please continue.)

☐ No (If you answered No, please stop here.)

[5] How often do you use a computer?

☐ Never

☐ Occasionally ☐ Once a month ☐ Twice a month ☐ Once a week ☐ Twice a week ☐ Every day or almost every day

Section Two: TAM Questions:

Questions	Strongly disagree	Disagree	Somewhat disagree	Neither agree or disagree	Somewhat agree	Agree	Strongly agree
The use of the Interactive Wall with Life Memories is a good recreation and leisure activity idea.	☐	☐	☐	☐	☐	☐	☐
I feel positive about the Interactive Wall with Life Memories.	☐	☐	☐	☐	☐	☐	☐
I am willing to continue using the Interactive Wall with Life Memories in the future.	☐	☐	☐	☐	☐	☐	☐
I intend to use the Interactive Wall with Life Memories frequently.	☐	☐	☐	☐	☐	☐	☐
I intend to use the Interactive Wall with Life Memories as a part of my everyday life.	☐	☐	☐	☐	☐	☐	☐
Using the Interactive Wall with Life Memories helps to recall my life memories.	☐	☐	☐	☐	☐	☐	☐
Using the Interactive Wall with Life Memories helps brain exercises and improves cognitive functions.	☐	☐	☐	☐	☐	☐	☐
Using the Interactive Wall with Life Memories improves my current physical condition and reminds me to take care of my own health situation.	☐	☐	☐	☐	☐	☐	☐
I feel that using the Interactive Wall with Life Memories is beneficial to my physical and cognitive health.	☐	☐	☐	☐	☐	☐	☐

I think I will play the Interactive Wall with Life Memories in the future without using the instructions.	☐	☐	☐	☐	☐	☐	☐
Learning how to use the Interactive Wall with Life Memories is easy for me.	☐	☐	☐	☐	☐	☐	☐
I find the Interactive Wall with Life Memories easy to learn.	☐	☐	☐	☐	☐	☐	☐
Interacting with the Interactive Wall with Life Memories was enjoyable.	☐	☐	☐	☐	☐	☐	☐
When interacting with the Interactive Wall with Life Memories, I had fun.	☐	☐	☐	☐	☐	☐	☐
Interacting with the Interactive Wall with Life Memories leads to exploration.	☐	☐	☐	☐	☐	☐	☐
I think that people who live in the village should try the Interactive Wall with Life Memories.	☐	☐	☐	☐	☐	☐	☐
I think that people who are important to me should use the Interactive Wall with Life Memories.	☐	☐	☐	☐	☐	☐	☐
I find that people who use the Interactive Wall with Life Memories are more likely to manage their health condition properly than those who do not use it.	☐	☐	☐	☐	☐	☐	☐
I think that the Interactive Wall with Life Memories is an innovative way to maintain good health.	☐	☐	☐	☐	☐	☐	☐
The quality of the Interactive Wall with Life Memories' graphical user interface design is high.	☐	☐	☐	☐	☐	☐	☐

The quality of the output I get from the Interactive Wall with Life Memories is high.	☐	☐	☐	☐	☐	☐	☐
I have no difficulty telling others about the results of using the Interactive Wall with Life Memories.	☐	☐	☐	☐	☐	☐	☐
I believe I could communicate to others the effects of using the Interactive Wall with Life Memories.	☐	☐	☐	☐	☐	☐	☐

Thank you for taking the time to participate in our survey. We truly value the information you have provided.

All your comments are welcome:
