# Supplementary material for: Acceptance of different design exergames in elders
Source: PLoS One. 2018 Jul 5;13(7):e0200185. doi: 10.1371/journal.pone.0200185 (PMC6033453; doi:10.1371/journal.pone.0200185)
Supplement: S6 File — (PDF) [file pone.0200185.s006.pdf]

Technology Acceptance Model Questionnaire of Ten Pretty Passes of the Bull.

Researcher:

Date:

Section One: Background Information:

[1] Gender ☐ Male ☐ Female

[2] Age: _______________ years old

[3] Educational Level:

☐ Elementary School ☐ Junior High ☐ Senior High ☐ Bachelor's Degree

☐ Graduate Degree

[4] Have you ever used a computer?

☐ Yes (If you answered Yes, please continue.)

☐ No (If you answered No, please stop here.)

[5] How often do you use a computer?

☐ Never

☐ Occasionally ☐ Once a month ☐ Twice a month ☐ Once a week ☐ Twice a week ☐ Every day or almost every day

Section Two: TAM Questions:

Questions	Strongly disagree	Disagree	Somewhat disagree	Neither agree or disagree	Somewhat agree	Agree	Strongly agree
The use of the Ten Pretty Passes of the Bull is a good recreation and leisure activity idea.	☐	☐	☐	☐	☐	☐	☐
I feel positive about the Ten Pretty Passes of the Bull.	☐	☐	☐	☐	☐	☐	☐
I am willing to continue using the Ten Pretty Passes of the Bull in the future.	☐	☐	☐	☐	☐	☐	☐
I intend to use the Ten Pretty Passes of the Bull frequently.	☐	☐	☐	☐	☐	☐	☐
I intend to use the Ten Pretty Passes of the Bull as a part of my everyday life.	☐	☐	☐	☐	☐	☐	☐
Using the Ten Pretty Passes of the Bull helps to recall my life memories.	☐	☐	☐	☐	☐	☐	☐
Using the Ten Pretty Passes of the Bull helps brain exercises and improves cognitive functions.	☐	☐	☐	☐	☐	☐	☐
Using the Ten Pretty Passes of the Bull improves my current physical condition and reminds me to take care of my own health situation.	☐	☐	☐	☐	☐	☐	☐
I feel that using the Ten Pretty Passes of the Bull is beneficial to my physical and cognitive health.	☐	☐	☐	☐	☐	☐	☐

I think I will play the Ten Pretty Passes of the Bull in the future without using the instructions.	☐	☐	☐	☐	☐	☐	☐
Learning how to use the Ten Pretty Passes of the Bull is easy for me.	☐	☐	☐	☐	☐	☐	☐
I find the Ten Pretty Passes of the Bull easy to learn.	☐	☐	☐	☐	☐	☐	☐
Interacting with the Ten Pretty Passes of the Bull was enjoyable.	☐	☐	☐	☐	☐	☐	☐
When interacting with the Ten Pretty Passes of the Bull, I had fun.	☐	☐	☐	☐	☐	☐	☐
Interacting with the Ten Pretty Passes of the Bull leads to exploration.	☐	☐	☐	☐	☐	☐	☐
I think that people who live in the village should try the Ten Pretty Passes of the Bull.	☐	☐	☐	☐	☐	☐	☐
I think that people who are important to me should use the Ten Pretty Passes of the Bull.	☐	☐	☐	☐	☐	☐	☐
I find that people who use the Ten Pretty Passes of the Bull are more likely to manage their health condition properly than those who do not use it.	☐	☐	☐	☐	☐	☐	☐
I think that the Ten Pretty Passes of the Bull is an innovative way to maintain good health.	☐	☐	☐	☐	☐	☐	☐
The quality of the Ten Pretty Passes of the Bull' graphical user interface design is high.	☐	☐	☐	☐	☐	☐	☐

The quality of the output I get from the Ten Pretty Passes of the Bull is high.	☐	☐	☐	☐	☐	☐	☐
I have no difficulty telling others about the results of using the Ten Pretty Passes of the Bull.	☐	☐	☐	☐	☐	☐	☐
I believe I could communicate to others the effects of using the Ten Pretty Passes of the Bull.	☐	☐	☐	☐	☐	☐	☐

Thank you for taking the time to participate in our survey. We truly value the information you have provided.

All your comments are welcome:
