# Supplementary material for: Acceptance of different design exergames in elders
Source: PLoS One. 2018 Jul 5;13(7):e0200185. doi: 10.1371/journal.pone.0200185 (PMC6033453; doi:10.1371/journal.pone.0200185)
Supplement: S11 File — (PDF) [file pone.0200185.s011.pdf]

Chang Gung Medical Foundation
Institutional Review Board

199, TUNG HWA NORTH ROAD,
TAIPEI, TAIWAN, 10507
REPUBLIC OF CHINA
Tel: (03) 3196200
Fax: (03) 3494549

Protocol Title：LES_Cloud - Advanced Eldercare Technology and Creative Space Construction

Protocol No. ：

IRB No. ：100-1075B

Principal Investigator(s) ：Dr. Kuam Wong, May-Kuen

Co-Investigator(s) ：Dr. Chia-Ying Chung; Dr. Yu-Cheng Pei

Duration of Approval: From 2011/08/01 TO 2015/06/30

Approved Protocol：2011/04/18 Ver1

Approved Informed Consent：2011/05/31 Ver2

Advertisement：None

Date of Meeting：2011/06/14

Date of Approval：2011/07/06

, was approved by the Institutional Review Board (the〝IRB〞) of Chang Gung Medical Foundation on （2011/07/06）. The IRB is organized and operates according to Good Clinical Practice and the applicable laws and regulations.

Sincerely Yours,

T. T. Hsieh

Tsang-Tang Hsieh, M.D.
Chairman
Institutional Review Board
Chang Gung Medical Foundation
