# Supplementary material for: Acceptance of different design exergames in elders
Source: PLoS One. 2018 Jul 5;13(7):e0200185. doi: 10.1371/journal.pone.0200185 (PMC6033453; doi:10.1371/journal.pone.0200185)
Supplement: S12 File — (PDF) [file pone.0200185.s012.pdf]

ClinicalTrials.gov PRS DRAFT Receipt (Working Version)
Last Update: 06/19/2017 22:42

ClinicalTrials.gov ID: NCT03084107

Study Identification

Unique Protocol ID: 100-1075B

Brief Title: Eldercare Technology in a Silver Culture Village

Official Title: LES_Cloud - Advanced Eldercare Technology and Creative Space

Secondary IDs:

Study Status

Record Verification: June 2017

Overall Status: Completed

Study Start: August 1, 2011 []

Primary Completion: June 30, 2015 [Actual]

Study Completion: June 30, 2015 [Actual]

Sponsor/Collaborators

Sponsor: Chang Gung Memorial Hospital

Responsible Party: Principal Investigator
Investigator: Alice May-Kuen Wong [walice]
Official Title: Attending Physician
Affiliation: Chang Gung Memorial Hospital

Collaborators:

Oversight

U.S. FDA-regulated Drug:

U.S. FDA-regulated Device:

U.S. FDA IND/IDE: No

Human Subjects Review: Board Status: Approved
Approval Number: 100-1075B
Board Name: Chang Gung Medical Foundation IRB
Board Affiliation: Chang Gung Memorial Hospital
Phone: +886-3-319-6200
Email: irb1@cgmh.org.tw
Address:

B2F., No.123, Dinghu Rd., Guishan Dist., Taoyuan City 333, Taiwan

Data Monitoring:

Study Description

Brief Summary: The aim of this project is to create ten smart devices that involve combining an interactive art environment with IC technology, arts and health administration. Investigators invited the elderly residents of Chang Gung Health and Culture Village to participate in the application of these devices, and participants were also requested to complete a technology acceptance model (TAM) questionnaire for these smart devices.

Detailed Description: According to statistics in Taiwan, by 2050, one-third population of Taiwan citizens will be over 65 years old. By the definition in World Health Organization, “Active aging” is the process of optimizing opportunities for health, participation and security in order to enhance the quality of life as people age. Active aging aims to extend healthy life expectancy and quality of life for all people as they age.

Chang Gung Health and Culture Village is one of the largest and most experienced aged care and retirement living providers in Taiwan.In this project, for promoting the successful aging of elderly residents, investigators will integrate health management systems and wireless sensor networks, physiological telemetry, learning and exercise behavior in Chang Gung Health and Culture Village. The investigators expect to build a Intelligent Care Facilities space for Elders base on exercise and rehabilitation with ten smart devices that involve combining an interactive art environment with computer science technology, multimedia applications and health administration in three years. Investigators invited the elderly residents to participate in the application of these devices, thereby increasing participants' physical activity and improving the physiological conditions. Participants were also requested to complete a technology acceptance model (TAM) questionnaire for these smart devices.

With respect to old age, long-term care is the human rights. How to provide an excellent eldercare to the elder people is a challenge. “Elder care”, “Technology”, “Art”, “Medicine”, a team of interdisciplinary experts is required to achieve this goal. The products of this study will promote an excellent elder care, in the setting of their choice by offering older people the information, services and support needed to make choices which enhance health, well-being and independence. In addition to analyze the acceptance model of intelligent care facilities for elders, the investigators expect the experience can help academic and industrial circles to research and develop care facilities for elders in the future.

Conditions

Conditions: Elderly

Keywords: Elder care
Active aging
Intelligent Care Facilities for Elders

Study Design

Study Type: Interventional

Primary Purpose: Other

Study Phase: N/A

Interventional Study Model: Single Group Assignment

Number of Arms: 1

Masking: No masking

Allocation: N/A

Enrollment: 39 [Actual]

Arms and Interventions

Arms	Assigned Interventions
Experimental: Questionnaire<br>technology acceptance model (TAM) questionnaire	Questionnaire<br>The elderly participated in the application of smart devices and requested to complete technology acceptance model (TAM) questionnaires for these devices.

Outcome Measures

Primary Outcome Measure:

1. TAM Questionnaire
   The Design Quality and Interface positively raised Awareness of Fun for the elderly participants in these smart devices, and the Perceived Usefulness positively affects the participants' willingness to use the devices.

   [Time Frame: 3 years]

Eligibility

Minimum Age: 60 Years

Maximum Age:

Sex: All

Gender Based:

Accepts Healthy Volunteers: Yes

Criteria: Inclusion Criteria:

- elderly who lived in the Chang Gung Health and Culture Village

Exclusion Criteria:

- inability to complete questionnaires

Contacts/Locations

Central Contact Person: Alice MK Wong, MD

Central Contact Backup:

Study Officials: Alice MK Wong
Study Principal Investigator
Chang Gung Memorial Hospital

Locations: Taiwan
Chang Gung Memorial Hospital

TaoYuan county, Taiwan, 333
Contact: Alice M.K. Wong, MD walice@adm.cgmh.org.tw

NOTE : TaoYuan county is not a recognized city for the specified state/country.

IPDSharing

Plan to Share IPD:

References

Citations:

Links:

Available IPD/Information:
